# Attapulgite Nanocomposites for Cartilage and Osteochondral Repair: Material–Tissue Matching, Evidence-Graded Mechanisms and Translation

**DOI:** 10.3390/nano16161021

**Published:** 2026-08-18

**Authors:** Junxu Zhu, Tao Shen, Siying Dong, Zongyan Cai, Wenhao Guo, Jiaxin Jin

**Affiliations:** Second Clinical Medical College, Lanzhou University, Lanzhou 730000, China; zhujx2024@lzu.edu.cn (J.Z.);

**Keywords:** attapulgite, palygorskite, cartilage repair, osteochondral interface, injectable hydrogel, nanoclay, material–tissue matching, drug delivery

## Abstract

Attapulgite (ATP; palygorskite) is a fibrous magnesium aluminum silicate that can reinforce hydrated polymer networks, provide a surface for molecular interactions, and participate in formulation-dependent ion or drug delivery. Although ATP has been studied most extensively in bone-oriented composites, its more distinctive role in cartilage repair may be as a spatially controlled regulator of the scaffold microenvironment rather than as a uniformly distributed bioactive filler. This review therefore examines ATP from a cartilage-first perspective. Direct ATP evidence, effects of modified ATP, performance of complete drug-loaded formulations, and cross-material extrapolations are considered separately. Current cartilage data support injectability, shear-thinning, photocrosslinking, mechanical reinforcement, and sustained intra-articular delivery but do not yet establish durable hyaline cartilage regeneration. In osteochondral constructs, ATP is more plausibly restricted to the calcified-cartilage interface or subchondral region, where reinforcement and mineral-associated functions may be beneficial, while high or uniform cartilage-side loading could increase stiffness, hypertrophy, or ectopic mineralization. This interpretation leads to testable design rules: define the ATP material fingerprint, map dose and spatial distribution, distinguish the true carrier phase, and assess cartilage matrix quality, lubrication, anti-hypertrophic stability, interface mechanics, persistence, and synovial safety. ATP should thus be developed as a dose-controlled and spatially restricted component whose value depends on material–tissue matching and direct mechanistic validation.

## 1. Introduction

Articular cartilage is avascular, sparsely cellular, and nourished largely by diffusion, leaving it with little capacity to restore a damaged surface [1]. Successful repair must reproduce a hydrated extracellular matrix, low friction, compressive resilience, and a stable chondrocyte phenotype without provoking hypertrophy or mineralization. These requirements differ from those of bone, where vascularization, mineral deposition, and increasing stiffness are usually desirable. The contrast becomes most important in osteochondral lesions: one construct must support cartilage at the surface, rebuild calcified cartilage at the interface, and permit vascularized subchondral bone formation below.

Attapulgite (ATP; palygorskite) offers several functions that could be useful in this setting. Its fibrous particles can reinforce hydrated polymers, while surface hydroxyl groups, grooves, and exchange sites can influence polymer bonding and the retention of selected molecules [2,3,4,5,6,7]. Yet, the same mineral character that supports a bone-side response may be undesirable in superficial cartilage if it produces excessive stiffness, persistent particles, or local mineralization. ATP should therefore be evaluated according to dose, surface state, carrier phase, and spatial placement rather than by its presence or absence alone. Because ATP also denotes adenosine triphosphate, the mineral name is written in full at first use.

The literature does not yet provide a simple answer. Direct cartilage studies are few, and many ATP formulations contain polymers, drugs, microspheres, doped ions, or additional minerals that can account for much of the measured response. Earlier reviews largely described nanoclay families or the structural and functional development of palygorskite [6,8,9]. They seldom asked whether ATP itself altered cartilage biology, whether it mainly improved the carrier network, or whether evidence from bone and other nanoclays had been extrapolated beyond its limits. This distinction is central because a favorable rheological result, short-term SRY-box transcription factor 9 (SOX9) expression, and restoration of a load-bearing hyaline surface represent very different levels of evidence.

This review uses material–tissue matching as its organizing principle. For cartilage, ATP should be judged by its effect on hydration, rheology, nutrient transport, matrix phenotype, lubrication, and hypertrophic stability. For the osteochondral interface, the questions shift to spatial mineralization, stress transfer, interlayer bonding, and subchondral remodeling. The resulting hypothesis is deliberately restrictive: ATP may be most useful at low cartilage-side doses or when concentrated toward the calcified interface and bone region, rather than when distributed uniformly through the construct.

The review begins with ATP structure and modification, then considers bone, cartilage, and the osteochondral interface in turn. Throughout, we distinguish the performance of a complete formulation from an ATP-specific effect, and a measured pathway from a plausible mechanism. This framing also brings the calcified interface and scaffold–host bond into the analysis, since both are easy to overlook when cartilage and bone are assessed separately.

Three evidence boundaries are maintained throughout. First, a property of an ATP-containing scaffold is not automatically an ATP-specific property. Second, osteogenic or angiogenic findings can inform subchondral design but cannot establish cartilage efficacy. Third, findings from laponite, halloysite, montmorillonite, sepiolite, or generic nanocomposite systems are used as design comparators rather than direct proof. By keeping these categories separate, the review aims to convert a limited and heterogeneous literature into testable cartilage-oriented design rules rather than a catalogue of positive material effects.

## 2. Material and Biological Basis of ATP for Cartilage and Osteochondral Engineering

### 2.1. Material Properties, Modification, and Cartilage-Relevant Functions

**Figure 1:**
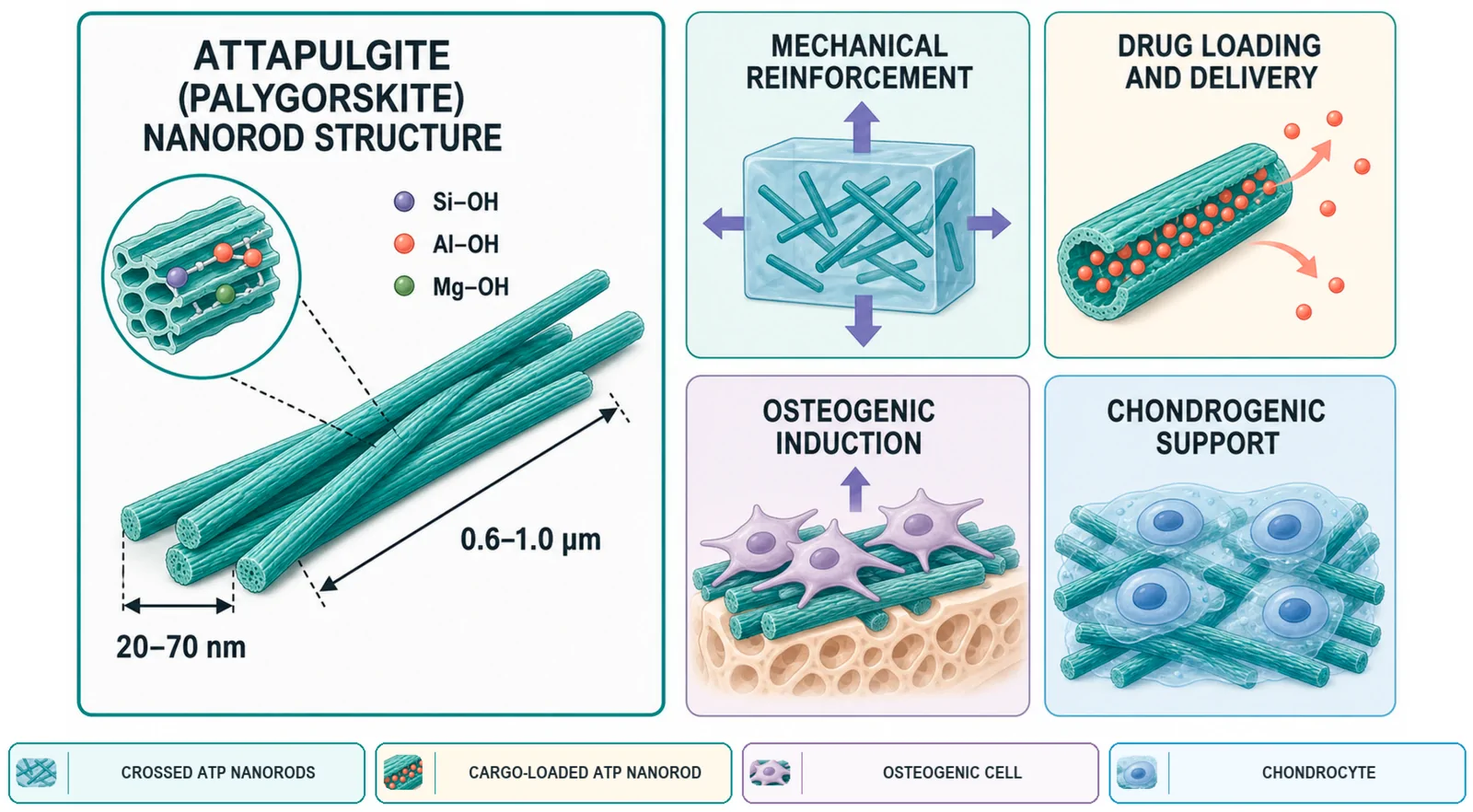
Structure-function map of attapulgite (ATP; palygorskite) nanorods and their proposed roles in biomaterial formulations. The left panel depicts the fibrous, channel-containing nanorod morphology, nominal dimensions of approximately 20–70 nm in diameter and 0.6–1.0 µm in length, and representative surface Si-OH, Al-OH, and Mg-OH groups. The four screenshots added beneath Figure 1 depict, from left to right, crossed ATP nanorods, a cargo-loaded ATP nanorod, an osteogenic cell, and a chondrocyte. These panels are conceptual. They do not establish that native ATP alone produces each biological effect, because polymer composition, ATP modification, therapeutic cargo, pore architecture, stiffness, and other formulation variables may contribute. ATP-free and property-matched controls are therefore required for ATP-specific attribution. Abbreviations: Al, aluminum; ATP, attapulgite; Mg, magnesium; Si, silicon.

Figure 1 links the principal structural features of ATP to the material functions discussed in the tissue-specific sections.

#### 2.1.1. Structure and Functional Properties

Palygorskite is a hydrated magnesium aluminum silicate built from alternating 2:1 silicate ribbons and chain-like units [6]. The resulting particles are rod-like, typically tens of nanometers in diameter and submicron to micron in length [2,6]. Their high aspect ratio is accompanied by Si-OH, Al-OH, and Mg-OH groups that participate in interfacial bonding, ion exchange, and adsorption [6]. Within a polymer scaffold, these rods can improve stress transfer and add physical crosslinks to hydrogel or fibrous networks [4,5].

Claims about drug loading need to identify both the cargo and the phase that actually carries it. Small polar molecules may reach accessible channels, exchange sites, grooves, or the outer mineral surface. Proteins and growth factors, by contrast, are more likely to remain on the external surface through hydrogen bonding, electrostatic or coordination interactions, or to be trapped in the surrounding polymer [4,6]. Thus, loading by an ATP-containing scaffold is not automatically loading by ATP. In the pioglitazone (PIO) and teriparatide (TPTD) examples discussed below, poly(lactic-co-glycolic acid) (PLGA) nanospheres or microspheres were the principal carriers [10,11].

Quantitative reporting is still uncommon. ATP-specific capacity should be given as cargo mass per mass of ATP, together with adsorption or encapsulation efficiency and recovery. Release data also benefit from comparison with zero-order, first-order, Higuchi, and Korsmeyer-Peppas models, provided that goodness-of-fit and mechanistic limits are reported. Desorption, diffusion, swelling, and polymer degradation may dominate at different times. Na-attapulgite tablets have been analyzed in this way [12], but an oral tablet is not a surrogate for a synovial cavity or bone defect. In musculoskeletal models, ATP residence time, nanorod clearance, and organ distribution remain undefined. The ATP/cyclodextrin pseudopolyrotaxane (PPR) formulation prolonged diclofenac delivery and anti-inflammatory activity, but did not establish an intra-articular half-life for ATP itself [4].

Short-term cytocompatibility and osteogenic activity are generally favorable, particularly in mesenchymal stem-cell experiments [5,7]. The longer view is much less certain. Persistence, biodistribution, degradation, and formulation-dependent ion exposure have not been characterized well enough to support claims of complete biodegradation or predictable clearance.

#### 2.1.2. Modification Techniques

Physical treatments mainly address dispersion. Heating removes adsorbed and zeolitic water from ATP channels and can alter surface area, porosity, and compatibility with a polymer matrix [6,13]. Grinding or sonication breaks up fiber bundles, although overly aggressive processing may shorten the rods and diminish their reinforcing effect [6]. Table 1 compares these approaches with chemical and ionic modification.

Surface chemistry offers a second route to control ATP behavior. Silane coupling agents introduce amino or carboxyl groups that strengthen polymer-mineral bonding [14], while glycine improves dispersion in organic matrices through interactions with surface hydroxyls [15]. In Sr-modified ATP hydrogels, osteogenic and angiogenic responses were associated with bone morphogenetic protein 2 (BMP2)/SMAD/runt-related transcription factor 2 (RUNX2) and phosphoinositide 3-kinase (PI3K)/protein kinase B (AKT)/hypoxia-inducible factor 1 alpha (HIF-1α)/vascular endothelial growth factor (VEGF) signaling [16]. These results are useful, but they describe a modified formulation. Comparisons with unmodified ATP and a clear dose–response remain essential.

### 2.2. Bone Evidence as Mechanistic and Subchondral Context

**Figure 2:**
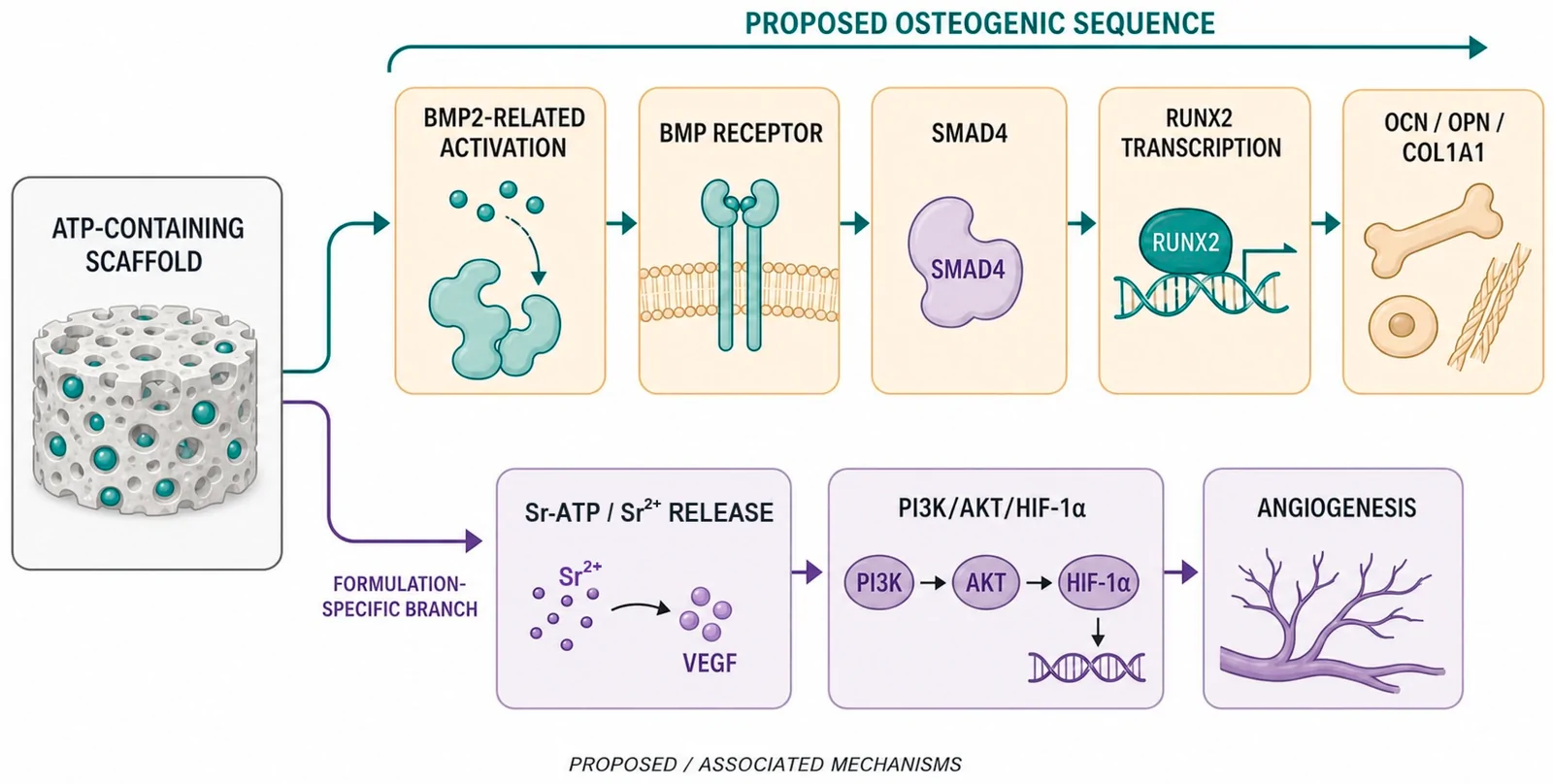
Proposed bone-oriented signaling sequences associated with selected ATP-containing formulations. The upper branch summarizes a BMP2-related sequence from BMP-receptor activation through SMAD4 and RUNX2 transcription to bone-associated markers, including OCN, OPN, and COL1A1. The lower, formulation-specific branch depicts Sr^2+^ release from strontium-modified ATP (Sr-ATP), VEGF-associated signaling through PI3K, AKT, and HIF-1α, and a proposed angiogenic response. The diagram synthesizes reported associations in complete bone-oriented formulations and is not a validated linear pathway map. It does not demonstrate that native ATP directly activates every node, nor should these bone findings be interpreted as evidence of cartilage regeneration. Potential contributions from strontium, therapeutic cargo, scaffold architecture, stiffness, and other formulation components require matched controls. Abbreviations: AKT, protein kinase B; ATP, attapulgite; BMP2, bone morphogenetic protein 2; COL1A1, collagen type I alpha 1 chain; HIF-1α, hypoxia-inducible factor 1 alpha; OCN, osteocalcin; OPN, osteopontin; PI3K, phosphoinositide 3-kinase; RUNX2, runt-related transcription factor 2; SMAD4, SMAD family member 4; Sr, strontium; VEGF, vascular endothelial growth factor.

Figure 2 summarizes bone-oriented pathways that may inform the subchondral compartment but should not be extrapolated directly to cartilage.

#### 2.2.1. What Bone Studies Reveal About ATP Formulations

Most ATP tissue-engineering work has focused on bone, where reinforcement, osteoconduction, and controlled ion exposure can all be advantageous. ATP-doped PLGA nanofibers improved human mesenchymal stem cell (hMSC) adhesion and osteogenic differentiation in association with BMP2/Smad/RUNX2 signaling [5]. Other studies placed ATP in polycaprolactone (PCL), PLGA, gelatin, or alginate matrices. A three-dimensional (3D)-printed gelatin/sodium alginate/nano-ATP hydrogel, for example, combined greater mechanical strength with interconnected pores [17], and biomimetic hydroxyapatite deposition on a printed nano-ATP/PCL scaffold improved osteoconductive performance in bone-defect models [18].

The carrier assignment is especially important in drug-loaded bone scaffolds. Fan et al. placed PIO-loaded PLGA nanospheres in a printed ATP/poly(vinyl alcohol) (PVA)/gelatin (GEL) matrix; PLGA carried the drug, while ATP contributed mainly to structure, rheology, and osteoconductive support [10]. No ATP-specific loading capacity in μg/mg can be derived from that study. Zhao et al. likewise loaded TPTD into PLGA microspheres before adding them to a GEL/PLGA/ATP scaffold [11]. Zhao et al. reported approximately 76% encapsulation efficiency and a pronounced early burst-release profile, indicating that release was not consistent with ATP-controlled zero-order delivery. Sr-ATP is different because the mineral itself was modified to release an ion. The strongest response was reported at 1.0% Sr-ATP and was associated with osteogenic and angiogenic signaling [16].

For a cartilage-centered interpretation, the main value of these bone studies is methodological. They show that ATP concentration, surface modification, polymer matrix, pore structure, and therapeutic cargo can change the response of the complete scaffold. They also reveal a recurring attribution problem: osteogenesis observed in a formulation containing Sr^2+^, PIO, TPTD, hydroxyapatite, or graphene oxide cannot be assigned to native ATP without matched controls. Most importantly, a material that increases mineralization in bone may increase hypertrophy or ectopic mineral deposition if used at the same dose in cartilage. Bone data should therefore guide the design of the subchondral module and the selection of controls, not serve as surrogate evidence for articular cartilage repair.

These studies establish preclinical promise, not clinical efficacy. Table 2 therefore presents the bone evidence by formulation and model rather than treating ATP as a single intervention.

#### 2.2.2. Limits of Extrapolating Bone Evidence to Cartilage

Several animal studies support a bone-repair effect. In rabbit radius defects, Col I/PCL/ATP scaffolds increased bone volume/total volume (BV/TV) at 12 weeks and upregulated Osterix, type I collagen (COL1), osteopontin (OPN), and alkaline phosphatase (ALP) [19]. Printed pure nano-ATP scaffolds also increased BV/TV and bone mineral density (BMD) in rabbit femoral condyle defects, with neovascularization around the implant at 8 weeks [2]. Sr-ATP hydrogels promoted bone formation and vascularization in rabbit calvarial defects [16]. The pattern is more consistent than in cartilage studies, although follow-up remains short and robust large-animal safety data are scarce.

### 2.3. ATP-Based Strategies for Cartilage Repair

**Figure 3:**
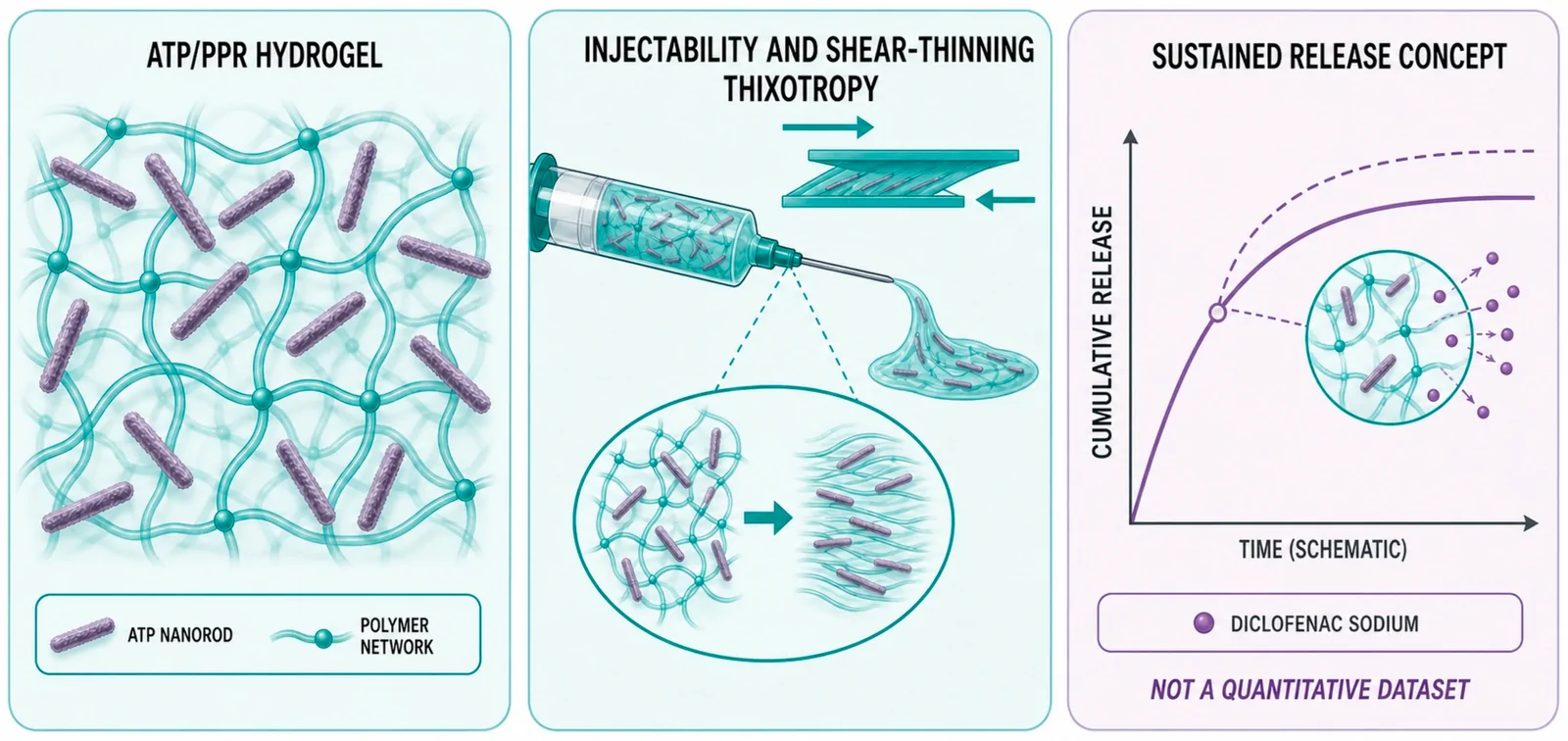
Conceptual material behavior of ATP/cyclodextrin pseudopolyrotaxane (PPR) supramolecular hydrogels. The left panel shows ATP nanorods dispersed within a polymer network, where nanorod-polymer interactions may contribute to physical reinforcement. The center panel illustrates syringe delivery, shear-induced network alignment or disruption during extrusion, and recovery after shear removal, which together represent injectability, shear-thinning behavior, and thixotropic recovery. The right panel presents a schematic cumulative-release concept for diclofenac sodium; the solid and dashed curves illustrate possible formulation-dependent profiles, and the inset depicts local release from the hydrogel network. The curves are not fitted experimental data and do not specify a release rate, kinetic model, encapsulation efficiency, or ATP-specific carrier contribution. Abbreviations: ATP, attapulgite; PPR, cyclodextrin pseudopolyrotaxane.

Figure 3 illustrates the material behavior reported for the ATP/PPR hydrogel.

#### 2.3.1. Injectable Hydrogels, Reinforcement, and Local Delivery

Cartilage applications are fewer and have focused mainly on reinforcement and intra-articular delivery. Ha et al. combined ATP with cyclodextrin pseudopolyrotaxane (PPR) to form an injectable supramolecular hydrogel [4]. The nanorods reinforced the network without eliminating shear-thinning or thixotropy. This is useful for local delivery in osteoarthritis or focal injury, but it is still a material performance result; durable hyaline cartilage regeneration has not yet been shown.

A cartilage scaffold must maintain water while allowing solute diffusion, withstand repeated compression without permanent collapse, and preserve a smooth low-friction surface. It must also support collagen II- and aggrecan-rich matrix deposition without shifting cells toward collagen type X alpha 1 chain (COL10A1)/matrix metalloproteinase 13 (MMP13)-associated hypertrophy. These functions are linked: increasing crosslink density may improve modulus but reduce nutrient transport, while adding a mineral nanofiller may strengthen the network but also alter swelling, surface roughness, and cell phenotype. For this reason, cartilage performance cannot be inferred from a single rheological or compressive measurement [1,20,21,22,23,24,25,26,27,28,29,30,31].

ATP may contribute at three levels. As a high-aspect-ratio particle, it can add physical crosslinks and improve stress transfer within a hydrogel. Its hydroxylated surface can interact with gelatin-, collagen-, or methacrylate-containing networks and may retain selected small molecules. Finally, the mineral surface and formulation-dependent Mg/Si exposure could alter cell behavior. The first two functions are supported more clearly than the third. None of them establishes that ATP directly activates a chondrogenic pathway, and each may become detrimental outside an appropriate concentration or spatial window [4,6,20,21,32].

The joint environment adds another constraint that is easy to miss in static cell culture. An injectable ATP formulation will encounter synovial proteins, inflammatory mediators, fluid exchange, and repeated shear almost immediately. Protein adsorption may alter particle dispersion and cellular recognition, while swelling or partial network degradation may expose nanorods that were initially immobilized. A useful cartilage study should therefore track rheology and release in synovial-like medium, quantify free and matrix-bound ATP over time, and relate these measurements to synovial-cell activation, macrophage behavior, and chondrocyte matrix production. Anti-inflammatory drug delivery may improve symptoms without restoring cartilage, so pain- or cytokine-related outcomes should be reported separately from structural regeneration [4,9,22,33,34,35,36,37,38,39,40,41,42,43,44,45,46].

Pore geometry may also steer local cell behavior. In a printed nano-ATP scaffold, pores near 200 μm were associated with a more hypoxic, cartilage-like environment, whereas pores near 500 μm favored osteogenic differentiation [2]. This result supports, but does not validate, a gradient-pore osteochondral design. Related composite studies found the strongest bone-forming response at 10% ATP in PCL/GEL/hydroxyapatite (HA) nanomembranes [47]. A 10% glycine-modified ATP (GATP)/poly(1,8-octanediol-co-citrate) (POC) scaffold also increased cartilage-associated collagen type II alpha 1 chain (COL2A1) and SOX9 together with bone-associated RUNX2 and osteocalcin (OCN) in a rabbit model [15].

The cartilage evidence is consequently promising but preliminary. Matrix quality, collagen II and aggrecan deposition, COL10A1/MMP13-mediated hypertrophy, integration, friction, and durability under repeated joint loading remain to be tested together.

#### 2.3.2. Chondrogenesis, Hypertrophy, and Functional Cartilage Endpoints

Photocrosslinking can stabilize an ATP-containing hydrogel after injection. In the ATP/PPR system, methacrylate crosslinking produced a more stable network with dispersed nanorods and a higher storage modulus. ATP may contribute physical crosslinks and hydrogen bonding with the photosensitive polymer. Mechanical matching alone, however, is not cartilage repair. The construct must preserve chondrocyte phenotype, allow nutrient transport, resist hypertrophic mineralization, and maintain a low-friction surface [20,21]. Possible effects of Mg- and Si-containing species on matrix synthesis remain plausible rather than established.

Photocrosslinking also introduces formulation-specific variables that require explicit reporting. Light wavelength, irradiance, exposure time, and photoinitiator concentration may affect polymer conversion, drug stability, and cell viability. ATP phase composition, surface chemistry, and dispersion should therefore be assessed before and after irradiation, together with drug recovery and bioactivity when a therapeutic cargo is present. Cell-laden systems additionally require viability, phenotype, and matrix-production measurements after crosslinking. Direct comparisons between ultraviolet- and visible-light protocols would help distinguish effects of irradiation from those of ATP, but these data remain limited in current ATP-containing cartilage formulations.

ATP/PPR hydrogels and related photocrosslinkable or drug-loaded composites may therefore serve as injectable cartilage-oriented platforms. Stronger regenerative claims will require long-term in vivo histology, biomechanics, anti-hypertrophic markers, and both local and systemic safety data. Table 3 summarizes the current formulations without implying that they are equivalent in evidence level.

The distinction between cartilage-associated markers and cartilage regeneration is particularly important. SOX9, COL2A1, and aggrecan (ACAN) can indicate a favorable early phenotype, but durable repair also requires abundant and appropriately organized matrix, a low collagen I fraction, suppression of COL10A1 and MMP13, resistance to calcification, and retention of mechanical function after repeated loading. Histology should be paired with biochemical glycosaminoglycan (GAG) analysis, indentation or confined-compression testing, friction and wear measurements, and integration with native cartilage. Where ATP is injected or can be released from a degrading network, synovial inflammation and particle persistence should be assessed in parallel [1,23,24,25,26,27,28,29,30,31,48,49,50,51,52,53,54,55,56,57,58].

A useful experimental design would compare a clay-free hydrogel, an ATP-containing hydrogel, a modulus-matched clay-free control, and, when ions or drugs are involved, soluble-ion or carrier-matched controls. ATP dose should be varied below and above the concentration that optimizes rheology. Spatial mapping of particles and matrix markers is preferable to reporting a bulk average, because a small mineral-rich region at the cartilage surface may have a different biological consequence from the same total ATP mass confined deeper in the construct. This approach would test whether ATP provides a genuine cartilage advantage or simply produces a stiffer formulation.

Carrier attribution is particularly important for intra-articular therapy. ATP may adsorb a small molecule on its outer surface or exchange sites, but a polymer microsphere, cyclodextrin assembly, or hydrogel mesh may control most of the observed retention. Loading capacity should therefore be reported separately for ATP and for the complete formulation, and release should be measured under joint-relevant agitation and protein content. A sustained concentration in the medium does not prove that ATP remained in the cartilage, nor does prolonged joint residence prove controlled cellular exposure. Fluorescent or elemental tracking, mass balance, and model-based release analysis would clarify which phase carries the drug and where that phase ultimately accumulates [4,10,11,36,59,60,61,62,63,64,65,66].

### 2.4. Cartilage-Centered Osteochondral Design and Interface Regeneration

**Figure 4:**
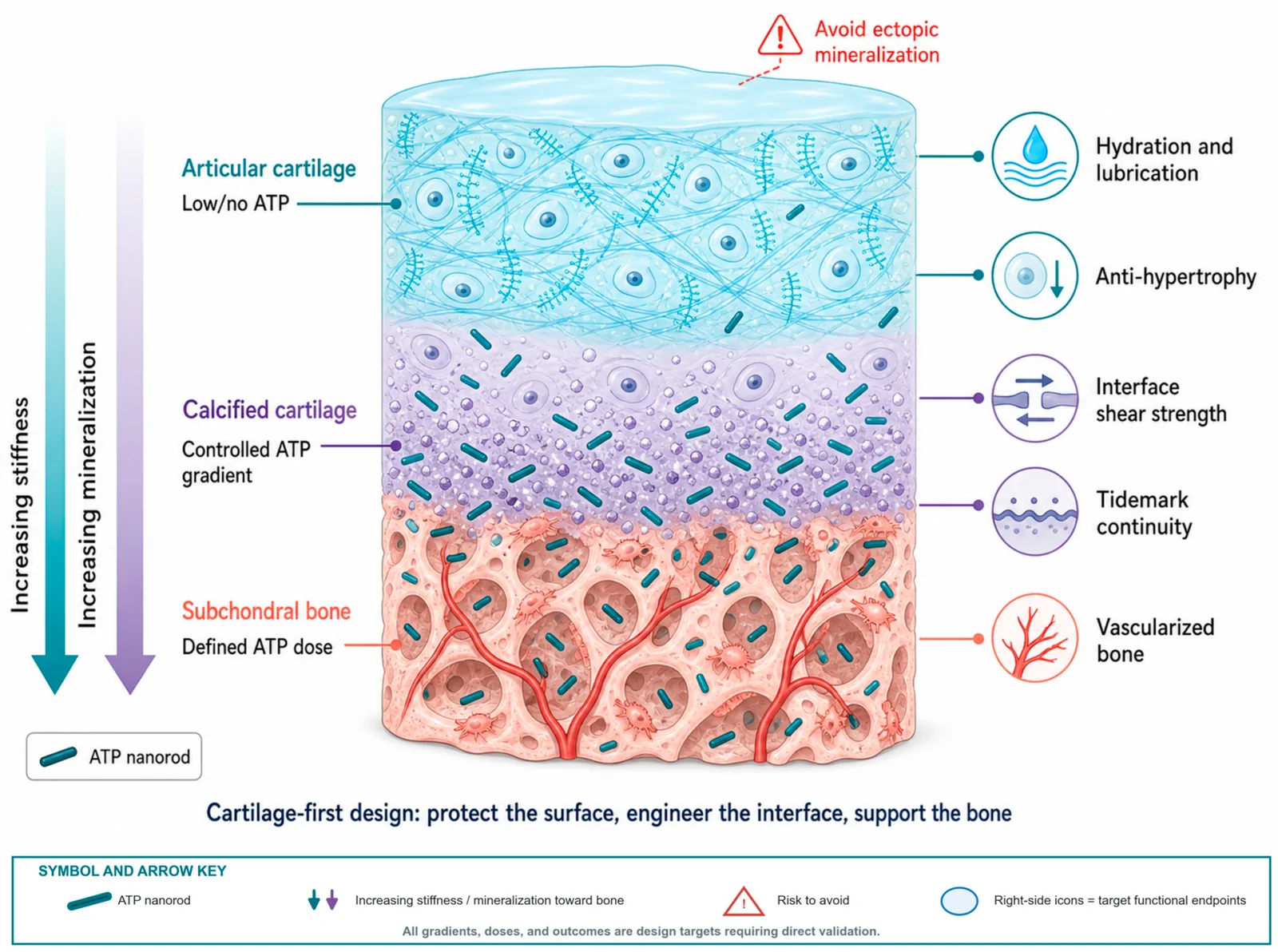
Cartilage-first spatial design targets for ATP-containing osteochondral constructs. The central schematic separates articular cartilage, calcified cartilage, and subchondral bone. Teal rods represent ATP nanorods; their illustrated density is qualitative and is not a measured concentration map. The proposed distribution maintains low or no ATP at the articulating cartilage surface, introduces a controlled ATP gradient across the calcified-cartilage interface, and uses a defined ATP dose on the bone side. The teal and purple downward arrows indicate proposed increases in stiffness and mineralization, respectively, from the articular surface toward subchondral bone. The red warning symbol identifies ectopic mineralization at the cartilage surface as a risk to avoid. The five right-hand icons denote target functional endpoints: hydration and lubrication, suppression of chondrocyte hypertrophy, interface shear strength, tidemark continuity, and vascularized bone formation. All spatial gradients, doses, and outcomes are design targets that require direct experimental validation. Abbreviation: ATP, attapulgite.

Figure 4 presents a cartilage-first zonal hypothesis in which ATP is restricted or graded toward the calcified interface and subchondral region; it is not a validated formulation.

The osteochondral unit is not simply cartilage placed on bone. Articular cartilage, calcified cartilage, and subchondral bone must transfer load while retaining different cell phenotypes and vascular environments. A useful construct would therefore combine a soft, anti-hypertrophic COL2A1/ACAN/SOX9-rich region, an interface that limits stress concentration and delamination, and a porous osteoconductive region that supports vascularization. In one rabbit study, a 10% GATP/POC scaffold increased COL2A1 and SOX9 on the cartilage side and ALP, mineralized nodules, RUNX2, and OCN on the bone side [15].

A cartilage-first design reverses the common tendency to optimize the bone phase and then add a softer upper layer. The superficial region should first be protected from excessive stiffness, mineral exposure, and vascular invasion. The interface can then be engineered as a narrow transition in which ATP content, pore size, and mineral nucleation increase gradually, followed by a more ATP-tolerant subchondral compartment. Such a distribution uses the same material differently across depth: low or absent ATP at the articulating surface, a controlled amount at the calcified interface, and a higher but still defined dose in the bone-side network [3,15,48,49,52,67,68,69,70].

Other ATP composites are relevant mainly as design clues. PCL/GEL/HA/ATP nanomembranes supplied a fibrous matrix and mineralization sites [47], while GO/ATP/COL scaffolds combined collagen matrix mimicry with graphene oxide- and ATP-associated osteogenic cues [71]. Both are largely bone-oriented. Neither is enough to demonstrate simultaneous cartilage restoration, interface formation, and subchondral remodeling in the same defect.

Mechanical and biological gradients have to be designed together. Cartilage needs hydration, low friction, and compressive resilience without unwanted mineralization. Subchondral bone needs greater stiffness, connected pores, vascular access, and mineral deposition. Between them, the interface must dissipate shear. ATP content, pore geometry, strand direction, interlayer bonding, and degradation rate are therefore linked variables. A very stiff bone layer may be counterproductive if it creates a sharp modulus jump or transfers damaging shear to new cartilage [48,49,52,67,68,69,70].

Calcified-cartilage reconstruction is a plausible but untested niche for ATP. Restricting nanorods to the interface might reinforce the region and provide controlled sites for mineral nucleation. The same design could also drive mineral encroachment, chondrocyte hypertrophy, or brittleness. Resolving this trade-off requires spatial measurements of ATP, mineral, tidemark continuity, calcified-cartilage thickness, and COL10A1/MMP13, rather than separate cartilage and bone markers.

This spatial hypothesis generates two competing predictions. Properly restricted ATP could strengthen the interface, reduce a sharp modulus discontinuity, and help organize a mineral gradient. Poorly controlled ATP could migrate toward cartilage, increase local roughness or stiffness, promote hypertrophic signaling, and blur the boundary between calcified and non-calcified cartilage. These alternatives can be distinguished by combining particle mapping with tidemark morphology, collagen II/I and COL10A1/MMP13 staining, mineral-density profiles, interface failure location, and synovial histology. The relevant question is therefore not simply whether ATP improves repair, but where it remains and which tissue response occurs at that location.

Mechanical integration should be followed over time rather than assessed only at implantation. A gradient that appears smooth in the manufactured scaffold may become discontinuous as the cartilage and bone polymers swell or degrade at different rates. Indentation mapping can reveal the evolving modulus profile, while push-out, pull-off, or lap-shear testing can identify whether failure occurs within new tissue, at an engineered interlayer, or at the host boundary. These measurements should be paired with gait or weight-bearing analysis because a histologically filled defect may still fail under joint loading. For ATP, the key mechanistic link is whether regional particle retention predicts improved interface strength without increasing superficial cartilage wear or mineralization [23,24,25,26,27,28,29,30,31,48,49,50,51,52,68,69,70].

Interface integration deserves its own endpoint. Push-out, pull-off, or lap-shear tests can identify bond strength and failure location; indentation mapping can reveal abrupt changes in modulus. These measurements should be paired with histological integration scores, calcified-cartilage continuity, and matched micro-computed tomography (micro-CT) and histology of subchondral remodeling. Otherwise, two well-formed but weakly attached tissues may be mistaken for an integrated repair.

PIO-, teriparatide-, and Sr-containing ATP formulations are informative for local delivery and bone-side signaling [10,11,16]. They do not, on their own, show coordinated regeneration of cartilage, calcified cartilage, and subchondral bone. Table 4 accordingly treats them as possible subchondral modules or comparators, rather than direct osteochondral evidence.

## 3. Critical Synthesis: A Cartilage-First Interpretation

Taken together, the literature supports a narrower and more useful interpretation of ATP. It is not a uniformly regenerative mineral and should not be judged only by whether its addition improves a composite. In cartilage-oriented systems, ATP is best viewed as a dose-dependent reinforcing, surface-active, and retention phase. In osteochondral systems, its most defensible niche is spatial: limited exposure in the superficial cartilage region and controlled enrichment toward the calcified interface or subchondral compartment. This interpretation separates scaffold performance from an ATP-specific mechanism and places cartilage protection ahead of bone-side mineralization.

### 3.1. Evidence Hierarchy and Boundaries of Cartilage Claims

Bone regeneration provides the most substantial ATP evidence. Studies in rodents and rabbits report osteogenic differentiation, higher RUNX2, ALP, OCN, OPN, or COL1, and gains in micro-CT measures such as BV/TV and BMD [2,5,10,11,16,19]. Even here, attribution is difficult. ATP content, pretreatment, porosity, polymer choice, and added agents differ across formulations, and ATP is often combined with strontium, teriparatide, pioglitazone, hydroxyapatite, graphene oxide, or growth-factor carriers. Better performance of the composite does not reveal which component caused it. Table 5 summarizes this evidence level and its remaining limits.

Cartilage evidence sits a step behind. Shear-thinning, injectability, thixotropy, in situ gelation, and sustained release are useful properties [4,20,21,32], but they are not equivalent to a durable hyaline matrix. The same caution applies to short-term SOX9 or COL2A1 expression. More persuasive studies would combine zonal matrix organization and GAG content with friction, compressive recovery, collagen II/I ratio, COL10A1/MMP13 suppression, integration, and preservation of subchondral bone.

Osteochondral work is conceptually advanced but experimentally uneven. Gradient and bilayer scaffolds fit the anatomy of the joint, yet cartilage-side and bone-side outcomes are still often reported separately [15,47,72]. Convincing repair would require chondrogenesis at the surface, a controlled mineral and mechanical transition through calcified cartilage, and vascularized osteogenesis below. Until these outcomes are shown in the same construct, dual-lineage claims should remain provisional.

SOX9 or COL2A1 demonstrate an early cartilage-associated response; histological filling demonstrates tissue formation; and low-friction, anti-hypertrophic, mechanically integrated repair demonstrates functional cartilage regeneration. ATP studies rarely reach the final level. Framing the evidence in these steps avoids both dismissing promising material behavior and overstating it as definitive cartilage repair.

### 3.2. Evidence-Graded Cartilage and Interface Mechanisms

Signaling data are frequently overinterpreted. BMP2/Smad/RUNX2 activity has been observed in ATP-containing osteogenic systems [5,10,16], and PI3K/AKT/HIF-1α/VEGF signaling in PIO- or Sr-containing formulations [10,16]. These findings support the tested composites, not a direct action of native ATP on every pathway. PIO, Sr^2+^, stiffness, pore structure, protein adsorption, and mineral surface chemistry remain entangled unless they are separated with matched controls.

A network of material–cell interactions is more credible than a single ATP-triggered cascade. Palygorskite nanorods expose channels, grooves, exchange sites, and hydroxylated surfaces [6]. These features can reinforce hydrated matrices and interact with collagen or gelatin, but direct comparisons with sepiolite, halloysite, or synthetic nanosilicates are lacking. Nor has hydroxyl density been quantitatively linked to fibronectin or BMP-2 adsorption. A protein corona and integrin engagement remain reasonable hypotheses, not established ATP-specific mechanisms.

Ion release needs the same restraint. Depending on composition, surface area, pH, and medium, palygorskite may release Mg^2+^ and soluble silicon species, but the concentrations around an implanted ATP scaffold are largely unknown. Biological diagrams should not depict dissolved silicon as free Si^4+^; soluble silicate or orthosilicic-acid species are more accurate. Evidence for Sr^2+^ signaling is stronger in Sr-ATP, yet that is a property of the modified material, not native ATP. Mechanical reinforcement, adsorption, ion exposure, cargo, and immune effects should be disentangled experimentally.

Mechanistic work will be most useful when material characterization and biological measurements share a time axis. Mineral phases, rod-length distribution, purity, surface charge and area, hydroxyl density, dispersion, ion speciation, release, mechanics, porosity, and degradation should be measured for the same formulation. Dose–response studies can then be paired with ion- and modulus-matched controls, pathway inhibition, macrophage co-culture, omics, and three-dimensional loading. Table 6 marks which links are supported, formulation-specific, or still hypothetical.

**Figure 5:**
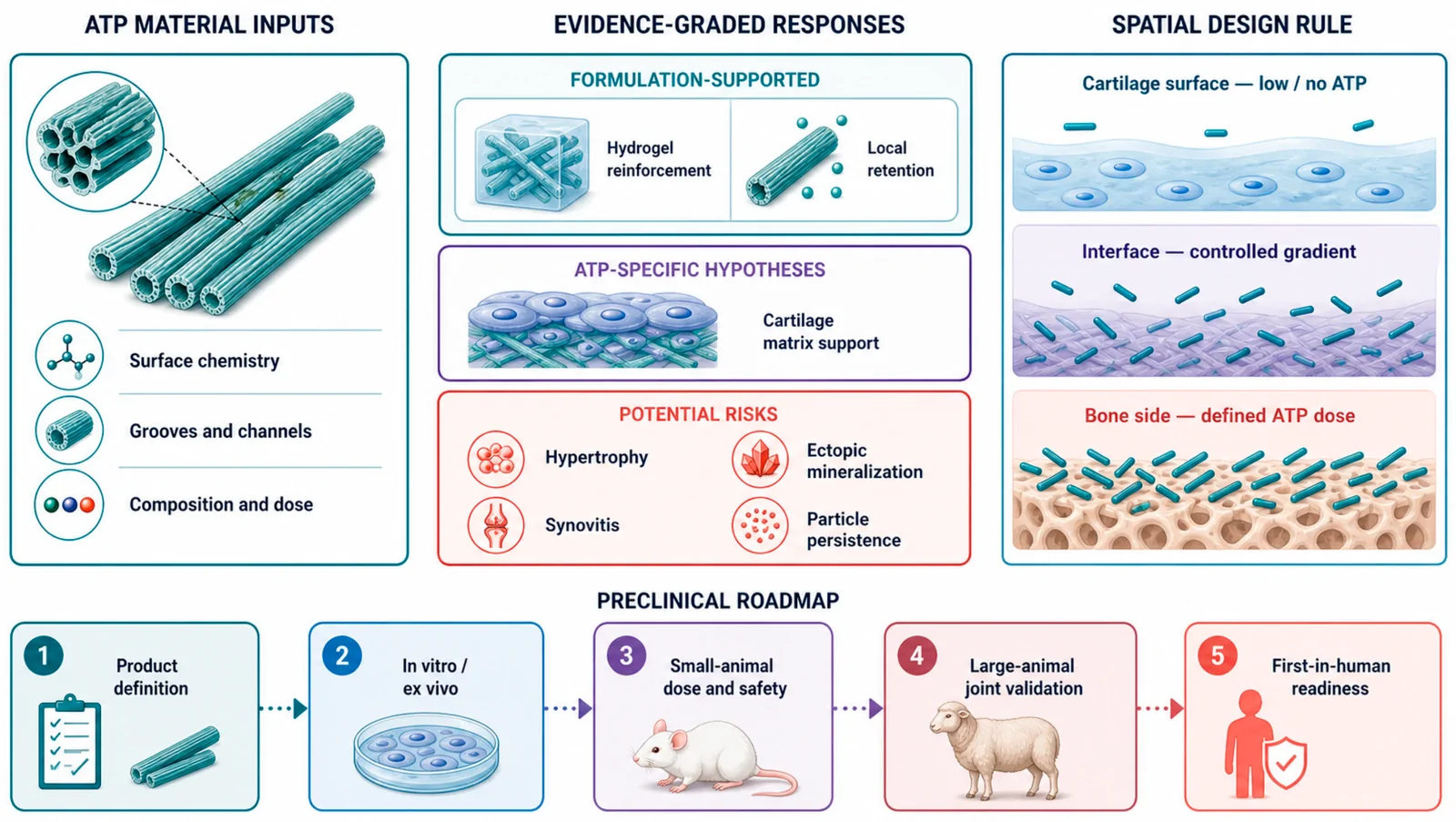
Integrated framework linking ATP material inputs, evidence-graded responses, spatial design, safety, and preclinical translation. The left column identifies three controllable material inputs: surface chemistry, grooves and channels, and composition and dose. The center column separates formulation-supported responses, including hydrogel reinforcement and local retention, from the ATP-specific hypothesis of cartilage-matrix support. It also lists four potential risks that should be measured alongside benefit: hypertrophy, ectopic mineralization, synovitis, and particle persistence. The right column applies a cartilage-first spatial rule, with low or no ATP at the cartilage surface, a controlled gradient at the interface, and a defined ATP dose on the bone side. The lower row presents five decision-gated stages: product definition, in vitro or ex vivo verification, small-animal dose and safety, large-animal joint validation, and first-in-human readiness. The categories and illustrations are conceptual; they indicate evidence boundaries and development priorities rather than quantitative effect sizes or validated clinical performance. Abbreviation: ATP, attapulgite.

Figure 5 consolidates the proposed ATP design logic into a single evidence-graded framework and links it to the translational sequence. Direct labels distinguish formulation-supported responses, ATP-specific hypotheses, and potential risks without relying on color-dependent arrow styles. The spatial-design panel aligns ATP exposure with tissue depth, while the lower row presents the five-stage roadmap summarized in Section 4.4.

### 3.3. Design Implications: ATP as a Spatially Restricted Cartilage-Interface Regulator

The first design implication is that ATP concentration should be treated as a regional variable rather than a single scaffold-wide percentage. Uniform loading is convenient for fabrication but biologically difficult to justify. The articulating cartilage surface is the compartment least tolerant of excess mineral, roughness, and stiffness, whereas the calcified interface and subchondral region may benefit from reinforcement and mineral-associated cues. A gradient formulation, layered printing strategy, or interface-bound particle phase is therefore more consistent with tissue physiology than homogeneous ATP distribution.

The second implication concerns mechanism. If ATP mainly reinforces the network, a modulus-matched ATP-free material should reproduce much of the response. If soluble Mg/Si species are responsible, an ion-matched control should do the same. If surface adsorption changes protein presentation, the effect should track ATP surface area, hydroxyl density, and the adsorbed protein corona. If none of these controls reproduces the response, an ATP-specific cellular mechanism becomes more credible. This sequence turns the proposed mechanism from a pathway diagram into a falsifiable set of experiments.

The third implication is that benefit and risk should be measured in the same experiment. Increased modulus should be interpreted alongside nutrient diffusion and friction; stronger mineralization alongside COL10A1/MMP13 and tidemark position; prolonged retention alongside synovitis and particle clearance. A formulation should advance only when the cartilage benefit remains after these competing outcomes are considered. This balance is the main conceptual contribution of a cartilage-first ATP framework.

These principles also provide a practical decision rule for formulation development. ATP should be retained only when it contributes an advantage that cannot be reproduced more simply by changing polymer concentration, crosslinking, pore geometry, or a clinically established carrier. If its benefit is confined to the bone side, the mineral should be excluded from the cartilage layer. If it improves local delivery, the active carrier phase and clearance route must be demonstrated. If a narrow interface dose improves load transfer without inducing hypertrophy, that regional use may justify the added manufacturing complexity. This decision-based approach prevents multifunctionality from becoming an end in itself and keeps cartilage quality as the criterion for material selection.

### 3.4. Application-Oriented Comparison with Other Nanoclays

No single nanoclay is optimal for every scaffold. ATP offers fibrous reinforcement, accessible grooves and channels, and a hydroxylated outer surface [6,8,73,74]. Sepiolite is also fibrous; halloysite provides a protected lumen; montmorillonite and bentonite offer expandable interlayers; laponite provides reproducible synthetic gelation; and kaolinite is inexpensive and readily modified. ATP therefore occupies a particular structure-function niche rather than owning a unique biological function. Table 7 compares these materials according to the intended cargo, tissue, and manufacturing route.

Current evidence supports further investigation of ATP at material interfaces rather than its general use as a universal drug carrier. Its channels and exchange sites suit some small polar species, while proteins mainly encounter the external surface or the surrounding polymer. Halloysite can protect suitable cargo within a lumen, and layered clays can favor intercalation. These are different design tools, not competing versions of the same mechanism. Laponite may also be preferable when highly reproducible hydrogel rheology is the main requirement. Meaningful comparison requires matched matrices, mineral dose, sterilization, cargo chemistry, and biological endpoints.

This interpretation is consistent with the wider nanomaterials literature. Across ATP, hydrogel, electrospun, and osteochondral systems, outcomes reflect the combined influence of mineral chemistry, matrix architecture, pore geometry, cargo, and interfacial bonding [9,22,23,24,25,26,27,28,29,30,31,34,35,36,37,38,39,48,49,50,51,52,53,54,55,56,57,58,75,76,77,78,79,80,81,82,83]. These studies are valuable comparators. Unless ATP itself was tested, however, they should guide design rather than be presented as ATP evidence.

Across this literature, osteogenesis is increasingly studied alongside inflammatory resolution and vascular remodeling. Ion-releasing and nanocomposite scaffolds can alter macrophage behavior, angiogenesis, and bone formation over different time windows [9,22,33,34,35,36,37,38,39,40,41,42,43,44,45,46,84,85,86,87,88,89,90,91,92,93,94,95]. ATP studies should capture those temporal relationships instead of relying on osteogenic markers alone. The comparator data help define controls and endpoints, but do not establish ATP-specific causality.

Delivery studies make a related point: loading and release depend on the cargo, particle geometry, polymer phase, and physiological medium. Work with ATP, halloysite, montmorillonite, sepiolite, and nanogels supports direct measurement of loading, desorption, residence time, and local toxicity rather than a generic assumption about clay carriers [36,59,60,61,62,63,64,65,66].

## 4. Translation and Future Priorities

### 4.1. Material Fingerprinting and Spatial Dose Control

A nominal ATP mass percentage does not define a cartilage formulation. Source, purity, rod-length distribution, surface area, charge, dispersion, dissolution, and sterilization may all alter the biological response. The material fingerprint should therefore be linked to spatial dose: total ATP mass, concentration in each layer, particle distribution, free-particle fraction after degradation, and release into synovial fluid or surrounding tissue [12]. Manufacturing specifications should include mixing or printing windows, interlayer fidelity, migration during swelling, storage stability, and post-sterilization rheology and surface chemistry.

### 4.2. Functional Cartilage and Interface Endpoints

Validation should begin with cartilage function and then extend through the interface. The superficial region requires collagen II- and aggrecan-rich matrix, low collagen I, suppression of COL10A1/MMP13, low friction, and recovery after repeated compression. The transition region requires a continuous tidemark, controlled mineral and modulus gradients, and resistance to shear or delamination. The subchondral region requires connected porosity, vascularized remodeling, and load transfer. ATP location should be mapped against all three outcomes, because a favorable bone response cannot compensate for a mineralized or poorly lubricated cartilage surface [2,17,68,69,70,96].

### 4.3. Safety and Regulatory Pathway

Regulatory planning is easier once the final formulation, route, and principal mode of action are fixed. An injectable intra-articular ATP hydrogel and an implanted osteochondral scaffold have different exposure and clearance profiles, and drug-, biologic-, or cell-containing systems may be combination products [97,98,99]. The International Agency for Research on Cancer (IARC) evaluation of fibrous palygorskite concerns inhalation hazard and cannot replace joint- or implant-specific testing [100]. A cartilage-focused safety package should include synovial inflammation, particle persistence and migration, macrophage and foreign-body responses, cartilage-surface wear, systemic organ burden, degradation, sterilization, shelf life, and large-animal joint function.

### 4.4. Preclinical Roadmap Toward First-in-Human Evaluation

A first-in-human program should be built around the final product rather than a generic ATP material. Injectable hydrogels, drug-device combinations, and implanted osteochondral scaffolds may require different evidence packages because their route, exposure, primary mode of action, and manufacturing risks differ. A practical sequence comprises five decision-gated stages. First, lock the product definition and analytical fingerprint, including ATP dose and spatial distribution. Second, use ATP-free and property-matched controls in vitro and ex vivo to demonstrate a cartilage- or interface-relevant functional benefit without cytotoxicity, hypertrophy, drug damage, or crosslinking-related confounding. Third, establish a dose and exposure window in a small-animal model while tracking synovitis, foreign-body responses, persistence, biodistribution, and systemic toxicity. Fourth, test the final formulation in a clinically relevant large-animal load-bearing osteochondral defect, with long-term evaluation of friction, zonal tissue quality, interface mechanics, subchondral remodeling, wear, and organ burden. Finally, demonstrate reproducible manufacturing, validated sterilization, packaging, shelf life, delivery, product classification, and early regulatory alignment before preparing a first-in-human protocol. Progression should require the decision gates summarized in Table 8 and Figure 5; isolated improvements in marker expression or bone formation are not sufficient.

## 5. Conclusions

ATP combines fibrous reinforcement, a hydroxylated surface, and formulation-dependent retention or mineral-associated functions. For cartilage repair, however, these attributes are enabling material properties rather than proof of direct chondrogenesis. The strongest current evidence concerns hydrogel reinforcement, injectability, and local delivery, while durable hyaline cartilage, lubrication, anti-hypertrophic stability, and long-term joint function remain insufficiently demonstrated.

The most productive development path is therefore cartilage-first and spatially controlled. ATP should be used at a defined dose, kept low or absent at the articulating surface, and enriched only where interface reinforcement or subchondral mineral-associated activity is required. Bone evidence can guide the lower compartment but should not be substituted for cartilage data. Progress toward translation will depend on the five-stage, decision-gated program defined here: product definition, in vitro/ex vivo verification, small-animal dose and safety, large-animal joint validation, and first-in-human readiness. Under this framework, ATP is not a universal regenerative filler; it is a testable cartilage microenvironment and osteochondral interface regulator whose benefit depends on material–tissue matching.

## Figures and Tables

**Table 1:** ATP modification techniques relevant to cartilage and osteochondral engineering.

Modification Technique	Mechanism	Advantages	Limitations	Representative Applications
Heat treatment	Removes adsorbed and zeolitic water, increases specific surface area and porosity	Simple operation, cost-effective; improves dispersion in polymer matrix	May alter crystal structure at excessive temperatures; requires temperature optimization	PLGA/ATP electrospun scaffolds for bone regeneration [5]
Mechanical grinding/ultrasonic dispersion	Breaks up ATP fiber bundles for uniform distribution in composites	Improved dispersibility; enhanced interfacial contact area	Risk of fiber damage or length reduction; energy-intensive process	Various ATP/polymer composite scaffolds
Silane coupling agent grafting	Covalently attaches functional groups (amino, carboxyl) to ATP surface	Strong interfacial bonding with polymer matrices; versatile surface functionalization	Complex reaction conditions; requires precise control of grafting density	Surface-functionalized ATP for scaffold reinforcement [14]
Amino acid modification (e.g., glycine)	Forms hydrogen bonds between amino groups and ATP surface hydroxyls	Markedly improved dispersion in organic polymers; enhanced biological activity	Limited to specific amino acid types; moderate bonding strength	GATP/POC scaffolds for osteochondral repair [15]
Ion doping (Sr^2+^, Mg^2+^)	Incorporates bioactive ions that activate cell signaling pathways	Dual osteogenic/angiogenic effects; sustained ion release profile	Narrow optimal concentration window; potential cytotoxicity at excessive doses	Sr-ATP hydrogel scaffolds for bone defect repair [16]

Abbreviations: ATP, attapulgite; GATP, glycine-modified attapulgite; Mg, magnesium; PLGA, poly(lactic-co-glycolic acid); POC, poly(1,8-octanediol-co-citrate); Sr, strontium.

**Table 2:** Bone-oriented ATP evidence relevant to subchondral scaffold design.

Scaffold Composition	Fabrication Method	Model/Duration	Key Outcomes	Ref
ATP/PLGA	Electrospinning	hMSC culture	Promoted adhesion and osteogenic differentiation via BMP2/Smad/RUNX2 pathway	[5]
Gelatin/Sodium alginate/nano-ATP	3D printing	In vitro	Improved hydrogel mechanical strength; porous network facilitated nutrient transport	[17]
nano-ATP/PCL + HA	3D printing + Biomimetic mineralization	Rat cranial defect	Significantly enhanced osteoconductivity	[18]
PIO-PLGA/ATP/PVA/GEL	Drug-loaded composite	BMSC culture	Sustained PIO release; activated BMP2/Smad/RUNX2 pathway; enhanced osteogenic differentiation	[10]
TPTD/PLGA/GEL/ATP	Microsphere-loaded composite	BMSC culture/Rabbit model	Controlled TPTD release over 8 weeks; upregulated COL1, RUNX2, OCN, OPN	[11]
Sr-ATP hydrogel	Hydrogel composite (Pickering emulsion)	Rabbit calvarial defect	1.0% Sr-ATP optimal; synergistic bone formation and vascularization via PI3K/AKT/HIF-1α/VEGF	[16]
Pure nano-ATP	3D bioprinting	Rabbit femoral condyle (8 wk)	Higher BV/TV and BMD; neovascularization around scaffold	[2]
Col I/PCL/ATP	Composite	Rabbit radius defect (12 wk)	Significantly higher BV/TV; upregulated Osterix, COL1, OPN, ALP	[19]

Abbreviations: ALP, alkaline phosphatase; ATP, attapulgite; BMD, bone mineral density; BMP2, bone morphogenetic protein 2; BMSC, bone marrow mesenchymal stem cell; BV/TV, bone volume/total volume; COL1, type I collagen; GEL, gelatin; HA, hydroxyapatite; HIF-1α, hypoxia-inducible factor 1 alpha; hMSC, human mesenchymal stem cell; OCN, osteocalcin; OPN, osteopontin; PCL, polycaprolactone; PI3K, phosphoinositide 3-kinase; PIO, pioglitazone; PLGA, poly(lactic-co-glycolic acid); PVA, poly(vinyl alcohol); RUNX2, runt-related transcription factor 2; TPTD, teriparatide; VEGF, vascular endothelial growth factor.

**Table 3:** Cartilage relevance and evidence boundaries of representative ATP-containing systems.

ATP System	Cartilage-Related Role	Current Evidence	Main Limitation	Ref.
ATP/PPR supramolecular hydrogel	Injectable reinforcement and local diclofenac delivery	Direct formulation evidence for shear-thinning, thixotropy, photocrosslinking, and sustained delivery	No proof of durable hyaline cartilage or ATP-specific residence time	[4]
Pure nano-ATP scaffold with pore-size control	Possible control of oxygen and lineage-related microenvironments	Smaller pores favored cartilage-associated conditions; larger pores favored osteogenesis	Primarily design-relevant and not a validated cartilage-repair formulation	[2]
GATP/POC integrated scaffold	Cartilage-side matrix support within an osteochondral construct	Early preclinical evidence for COL2A1/SOX9 together with bone-side osteogenic markers	Long-term cartilage quality, hypertrophy, friction, and interface mechanics remain unclear	[15]
Photocrosslinkable ATP-containing network	In situ stabilization and mechanical tuning	Hydrogen-bonding and physical-crosslinking rationale with cartilage-relevant modulus	Mechanics alone do not establish chondrogenesis or functional repair	[20,21]
PCL/GEL/HA/ATP nanomembrane	Fibrous matrix comparator for a possible mineralized interface	Strongest response at an intermediate ATP concentration	Evidence is predominantly bone-oriented and should not be used as direct cartilage proof	[47]
Mg/Si-related ATP hypothesis	Potential matrix or cell-response modulation	Biologically plausible based on ion-responsive biomaterials	Local speciation, dose, cartilage benefit, and hypertrophy risk are not established	[20,32]

Abbreviations: ATP, attapulgite; COL2A1, collagen type II alpha 1 chain; GATP, glycine-modified attapulgite; GEL, gelatin; HA, hydroxyapatite; Mg, magnesium; PCL, polycaprolactone; POC, poly(1,8-octanediol-co-citrate); PPR, cyclodextrin pseudopolyrotaxane; Si, silicon; SOX9, SRY-box transcription factor 9.

**Table 4:** Direct, indirect, and compartment-specific evidence for ATP in cartilage-centered osteochondral repair.

ATP System	Evidence Context	Osteochondral Relevance	Evidence Boundary	Ref
GATP/POC integrated scaffold	Rabbit osteochondral model; dual-lineage markers	Direct early preclinical evidence for coordinated cartilage- and bone-side responses	Interface mechanics, calcified-cartilage restoration, and long-term durability remain unresolved	[15]
Pure nano-ATP scaffold with pore-size control	Bone-defect model and pore-dependent lineage observations	Supports gradient-pore design and spatial control of ATP	Indirect design evidence; not a validated full osteochondral construct	[2]
ATP/PPR injectable hydrogel	Cartilage-oriented rheology and delivery platform	Potential superficial cartilage layer or local delivery component	No integrated osteochondral validation	[4]
PIO-, teriparatide-, or Sr-containing ATP formulations	Bone-oriented delivery and osteogenic/angiogenic studies	Potential subchondral-bone module in a zonal construct	Formulation evidence only; not direct cartilage or interface proof	[10,11,16]

Abbreviations: ATP, attapulgite; GATP, glycine-modified attapulgite; PIO, pioglitazone; POC, poly(1,8-octanediol-co-citrate); PPR, cyclodextrin pseudopolyrotaxane; Sr, strontium.

**Table 5:** Evidence hierarchy for a cartilage-centered interpretation of ATP biomaterials.

Application	Evidence Level	Supported Functions	Key Gaps Before Stronger Claims
Cartilage repair	Early-stage	Injectable and thixotropic networks; mechanical reinforcement; local delivery; short-term cartilage-associated markers.	Hyaline matrix quality; anti-hypertrophic stability; lubrication and wear; integration; long-term joint models.
Osteochondral repair	Conceptual to early preclinical	Cartilage-first zonal design; pore and mineral gradients; potential interface-restricted ATP placement.	Calcified-cartilage reconstruction; continuous mechanical integration; durable load-bearing function.
Bone evidence used as context	Moderate preclinical	Reinforcement, osteoconduction, osteogenic markers, and bone formation in selected defects.	Cannot be extrapolated directly to superficial cartilage; component attribution and long-term persistence remain unresolved.
Clinical translation	No mature clinical evidence	Feasibility of ATP-containing cartilage- and bone-oriented formulations.	Material fingerprinting; intra-articular clearance; chronic synovial safety; GMP manufacturing and regulatory classification.

Abbreviations: ATP, attapulgite; GMP, good manufacturing practice.

**Table 6:** Evidence-graded mechanism and risk map for ATP in cartilage and osteochondral repair.

ATP Feature	Proposed Material Effect	Putative Biological Consequence	Current Evidence Status	Required Validation
High-aspect-ratio fibrous nanorods	Reinforcement, physical crosslinking, and increased matrix-filler contact	Altered cell adhesion and mechanotransduction; improved load transfer	Material reinforcement is supported; ATP-specific mechanotransduction remains indirect	Matched-modulus controls, dispersion mapping, and pathway inhibition under 3D loading
Channels, grooves, exchange sites, and external surface	Adsorption or ion exchange for selected small molecules; surface retention of larger cargo	Localized exposure and slower release when combined with a polymer network	Supported in pharmaceutical formulations; musculoskeletal studies often confound ATP with PLGA or hydrogel carriers	Report μg cargo per mg ATP, encapsulation efficiency, desorption, and model-fitted release kinetics
Si-OH, Mg-OH, and Al-OH-rich surface	Hydrogen bonding, electrostatic interactions, and protein-corona formation	Potential integrin-mediated adhesion and lineage support	Plausible, but ATP hydroxyl density has not been quantitatively linked to fibronectin or BMP-2 adsorption	Hydroxyl-density titration, quantitative proteomics, adsorption isotherms, and receptor-blocking studies
Mg-bearing aluminosilicate framework	Release of Mg^2+^ and soluble Si species under formulation-dependent conditions	Potential osteogenic or angiogenic support	Biologically plausible; local concentrations and physiological relevance are not established for ATP scaffolds	Speciation-aware release testing in physiological media, dose–response windows, and ion-matched controls
Exchangeable or deliberately doped ions (e.g., Sr^2+^)	Controlled ion delivery from a defined modified formulation	BMP2/Smad/RUNX2- and PI3K/AKT/HIF-1α/VEGF-associated responses	Formulation-specific evidence is available for Sr-ATP; it should not be generalized to native ATP	Unmodified ATP, soluble-ion, and equal-mechanics controls with time-resolved signaling
Natural-source mineral and variable fiber-length distribution	Batch-dependent purity, surface area, dissolution, and persistence	Variable efficacy and potential macrophage/foreign-body responses	Source variability is established; long-term implant-specific safety evidence remains limited	Mineral phase quantification, fiber-length specification, impurity/endotoxin limits, biodistribution, and chronic histology

Abbreviations: ATP, attapulgite; BMP2, bone morphogenetic protein 2; HIF-1α, hypoxia-inducible factor 1 alpha; Mg, magnesium; PI3K, phosphoinositide 3-kinase; PLGA, poly(lactic-co-glycolic acid); RUNX2, runt-related transcription factor 2; Si, silicon; Sr, strontium; VEGF, vascular endothelial growth factor.

**Table 7:** Application-oriented comparison of attapulgite with representative nanoclays used in tissue engineering.

Nanoclay	Dominant Structure	Main Advantage	Main Limitation	Evidence-Aligned Potential Application Context
Attapulgite/palygorskite	One-dimensional fibrous nanorods with surface hydroxyl groups	Fibrous reinforcement, accessible channels/grooves, surface-mediated retention, and mineral-like interface construction	Natural-source variability; cargo-dependent adsorption; uncertain long-term persistence and ion-release safety	Candidate use in bone-oriented or spatially zoned osteochondral composites requiring reinforcement; direct cartilage benefit and long-term safety remain to be validated
Bentonite/montmorillonite	Layered two-dimensional silicate	Interlayer adsorption and swelling-assisted drug loading	Weaker fiber-like reinforcement; swelling may alter scaffold stability	Drug-delivery systems or barrier-like composite matrices
Halloysite nanotubes	Hollow tubular one-dimensional particles	Protected lumen encapsulation and staged release for suitably sized small-molecule cargo	Cargo size and loading method limit lumen access; less suited to some surface-mediated or macromolecular loading strategies	Controlled delivery where lumen loading is the dominant design objective
Kaolinite	Layered aluminosilicate sheets	Low cost, availability, and ease of surface modification	Limited mechanical reinforcement and relatively low surface area	Large-scale or low-cost composite applications after adequate purification
Laponite	Synthetic nanosilicate discs	High purity, reproducible gelation, and strong hydrogel interaction	Higher cost and regulatory uncertainty for some formulations	Injectable hydrogels, bioinks, and systems requiring reproducible nanosilicate behavior

**Table 8:** Staged preclinical roadmap and decision gates for advancing ATP-based cartilage and osteochondral biomaterials toward first-in-human evaluation.

Stage	Primary Question	Minimum Evidence Package and Decision Gate
1. Product definition	What exactly is the product and intended clinical use?	Define the final formulation, route, primary mode of action, ATP phase fraction, accessory minerals, elemental impurities, endotoxin, particle-size distribution, surface charge, dissolution, spatial dose, sterilization, and shelf life. Gate: reproducible release specifications across batches.
2. In vitro and ex vivo verification	Does ATP add a cartilage- or interface-relevant benefit without introducing avoidable harm?	Use ATP-free and modulus-matched controls; quantify cytocompatibility, chondrocyte phenotype, glycosaminoglycan and collagen matrix, hypertrophy, friction, compression, ATP dispersion or migration, and drug recovery after crosslinking. Gate: predefined functional benefit without increased cytotoxicity or hypertrophy.
3. Small-animal dose and safety	What dose and exposure window are locally and systemically tolerable?	Establish dose response, local synovitis and foreign-body reactions, persistence, biodistribution, and early interface integration. Gate: a justified exposure window with no dose-limiting local or systemic toxicity.
4. Large-animal joint validation	Does the final construct provide durable function in a clinically relevant load-bearing defect?	Use an orthotopic osteochondral model and an ATP-free comparator; assess long-term weight bearing, friction, zonal histology, interface mechanics, micro-computed tomography, wear, synovium, and organ burden. Gate: reproducible functional superiority with acceptable safety.
5. First-in-human readiness	Can the locked product be manufactured, delivered, and regulated consistently?	Establish good manufacturing practice-compatible production, validated sterilization, packaging, shelf life, delivery procedure, product classification, and early regulator alignment. Gate: a locked product and risk-based evidence package suitable for a first-in-human protocol.

Abbreviations: ATP, attapulgite. The evidence package should be adapted to the final product, intended use, route of administration, and primary mode of action.

## Data Availability

No new datasets were generated or analyzed in this narrative review. The evidence discussed is available in the cited publications.

## Abbreviations

ACAN	aggrecan
AKT	protein kinase B
ALP	alkaline phosphatase
ATP	attapulgite
BMD	bone mineral density
BMP2	bone morphogenetic protein 2
BMSC	bone marrow mesenchymal stem cell
BV/TV	bone volume/total volume
COL1	type I collagen
COL1A1	collagen type I alpha 1 chain
COL2A1	collagen type II alpha 1 chain
COL10A1	collagen type X alpha 1 chain
EMA	European Medicines Agency
EtO	ethylene oxide
FDA	U.S. Food and Drug Administration
GAG	glycosaminoglycan
GATP	glycine-modified attapulgite
GEL	gelatin
GMP	good manufacturing practice
GO	graphene oxide
HA	hydroxyapatite
HIF-1α	hypoxia-inducible factor 1 alpha
hMSC	human mesenchymal stem cell
IARC	International Agency for Research on Cancer
MMP13	matrix metalloproteinase 13
micro-CT	micro-computed tomography
OCN	osteocalcin
OPN	osteopontin
PCL	polycaprolactone
PI3K	phosphoinositide 3-kinase
PIO	pioglitazone
PLGA	poly(lactic-co-glycolic acid)
POC	poly(1,8-octanediol-co-citrate)
PPR	cyclodextrin pseudopolyrotaxane
PVA	poly(vinyl alcohol)
RUNX2	runt-related transcription factor 2
SMAD4	SMAD family member 4
SOX9	SRY-box transcription factor 9
TPTD	teriparatide
VEGF	vascular endothelial growth factor
